# Marine Natural Products: A Way to New Drugs

**Published:** 2009-07

**Authors:** V.A. Stonik

**Affiliations:** 1 Pacific Institute of Bioorganic Chemistry, Far-eastern Branch of the Russian Academy of Science

## Abstract

The investigation of marine natural products (low molecular weight bioregulators) is a rapidly developing scientific field at the intersection of biology and chemistry. Investigations aimed at detecting, identifying, and understanding the structure of marine natural products have led to the discovery of 20,000 new substances, including those characterized by an extremely high physiological activity. Some results and prospects of works aimed at creating new drugs on the basis of marine natural products are discussed herein.

## Introduction 

Low molecular natural products are largely referred to the so-called secondary metabolites. In contrast to primary metabolites, these substances are rare in occurrence and may be detected only in some taxa, and occasionally, in one biological species (subspecies, strain). They are formed on the basis of precursor substances participating in primary metabolism, such as acetic acid, amino acids, glucose and are observed mainly as final products of biochemical transformations. Secondary metabolites are quite diverse by chemical structure and include steroids, terpenoids, alkaloids, polyketides, phenolic metabolites, some carbohydrates, lipids, and peptides. On the other hand, secondary metabolites can be classified on the basis of their biological functions as hormones, antibiotics, toxins, pheromones, etc. Among the natural products there are endo-metabolites, i.e., substances exercising their biological functions in the organisms-producers, for instance, oxylipins, hormones, phytoalexins, and more numerous exometabolites released into the environment and being of ecological importance, including toxins, antibiotics, and different signal compounds.

The higher terrestrial plants and soil microorganisms were, for a long time, considered to be the major biological sources of natural products. However, when skin-diver equipment was invented and became widely used, different marine organisms began also to be referred to their sources. The study of marine organisms significantly increased the amount of known natural products. In fact, the total number of studied natural products is unlikely to exceed 120-150 thousands, and by the estimates of many scientists, the amount is even lower [1], whereas about 20 thousand natural substances have been segregated. Moreover, the first researchers were surprised by the fact that marine organisms very rarely contained already known compounds. Hence, the biochemistry of their secondary metabolism differs substantially from that of terrestrial organisms. This fact may be explained by significant taxonomic differences between terrestrial and marine animals, plants and microorganisms.

As a whole, the investigation of natural products is of ecological importance. It stimulates the development of organic synthesis, physicochemical and isolation methods, as well as other sciences, such as biochemistry, molecular genetics, biotechnology, and microbiology. Moreover, it is closely related to healthy diet.

Natural products have played and continue to play an important role in the creation of new drugs and development of the pharmaceutical industry around the world. Analgesic preparations based on morphia from opium, cardioactive digitalis glycosides, anti-inflammatory agents created in the course of the investigation of steroid hormones, antibiotics, and many others are included in a list of products developed on the basis of natural drugs or their derivatives and analogues, which contains about 50% of all currently known medical products [5].

Results of the investigation of marine natural products which have been used or are being used now for the creation of new drugs are considered herein.

## Marine Natural Products with Antitumor Properties.

**Fig. 1.**
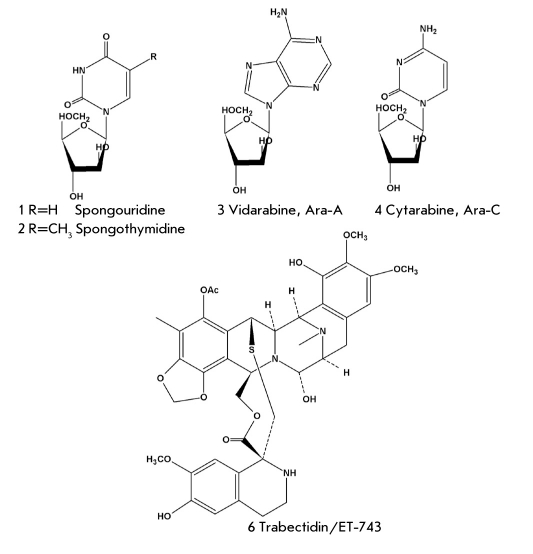
Antitumor and antiviral drugs created on the basis of marine natural products.

With the use of an aqualung, natural-product chemists got a chance to study more and more marine organisms. The American scientist Werner Bergmann was one of the first to start their chemical investigation. In 1951, he reported the isolation of unusual nucleosides (spongothymidine (1) and spongouridine (2) (Fig. 1), and then others) [2-4] from the sponge Cryptotethia crypta collected near the coast of Florida. They contained arabinose residues, instead of the ribose and desoxyribose residues observed in most compounds of that class. Those investigations stimulated the appearance of the antimetabolite conception in pharmacology. Antimetabolites are the active substances of drugs, which are characterized by a significant similarity to, and structural difference from, human metabolites. Antimetabolites participate in the biosynthesis of some biopolymers, more often, of DNA, and inhibit its exhibiting antitumor and antiviral properties. Bergmann's discovery was followed by the development of two arabinonucleoside drugs: arabinoadenine (3) (ara-A, Vidarabine) and arabinocytosine (4) (Ara-C, Cytarabine) (Fig. 1), which were used in clinical practice as antitumor and antiviral drugs for tens of years. Several other drugs of a nucleoside nature (azidothymidine, acyclovir, etc.) differ from ordinary nucleosides in other structural features. For instance, azidothymidine has an azide group in its monosaccharide residue, while acyclovir is characterized by an open furanose cycle.

**Fig. 2.**
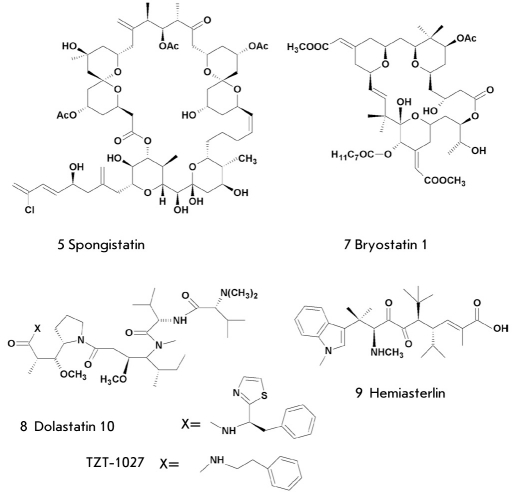
Some compounds tested as active substances of antitumor drugs.

However, further development of antitumor drugs on the basis of marine natural products was not just so successful. The case was not that there were no compounds with high antitumor activity. On the contrary, some marine invertebrates had minor secondary metabolites characterized by extremely high toxicity against tumor cells. By their cytotoxicity, they are hundreds and thousands of times superior to most active antitumor drugs currently in use. For instance, spongistatin (5) (Fig. 2) from marine tropic sponges is the most active of all natural and synthetic compounds found over the history of the investigation of antitumor compounds at the National Institute of Cancer (USA). It was initially found in one of the sponges, owing to the biological activity of the corresponding extracts, but for a long time the compound itself could not be obtained in the amount required for a structural investigation. Only after the collection and processing of three tones of sponge did the scientists finally manage to obtain 0.8 mg of spongistatin. Then, another sponge collected near the Maldive Islands was used as a basic material, and after the processing of 400 kg of this sponge, the scientists obtained another 10 mg of spongistatin, shed light on its structure, and began analyzing the physiological action of that macrolide. The inhibitory dose needed to cause the death of 50% of tumor cells (IC_50_) was 10^-10^ M for colon cancer cells and 10^-12^ M for breast cancer cells. In experiments on animals with deadly malignant tumors, 70% of them survived after the injection of 25 μg/kg of spongistatin.

As a whole, several dozens of marine metabolites, extremely toxic against tumor cells, were detected. It is very important that many of them belong to the fundamentally new structural series of antitumor substances that opens good prospects for synthetic modeling. Until those substances were discovered, all of the natural products applied in chemotherapy were referred to not more than 4-5 structural types.

However, the creation of new-generation medical products based on marine natural products is being slowed down by some complicated issues. Firstly, these substances are difficult to access. It is impossible to harvest a sufficient amount of these substances from marine organisms, because their producers, as a rule, are rare and disseminated species, while the methods for aquaculture of such biological products remain poorly developed. Economically, reasonable syntheses for most of these substances have yet to be developed as well, due to the complexity of their structures and abundance of asymmetric centres in them. Secondly, these compounds, highly toxic against tumor cells, do not always demonstrate good anticancer activity when we are dealing with people or animals. And, thirdly, some of them are characterized by side effects; for instance, they can negatively affect kidneys or other organs and systems, which excludes their clinical application.

Nevertheless, development of another antitumor drug based on marine natural products was successfully completed just recently. In the end of 2007, the drug trabectidin (Yondelis) was approved in European Union countries for treatment of soft tissue sarcoma. The structure of the corresponding active substance, alkaloid ecteinascidin-743 (ET -743) (6) (Fig. 1) from the ascidian Ecteinascidia turbinata, was elucidated independently by two groups of American scientists 18 years ago [7, 8]. However, the high antitumor activity of extract E. Turbinate had been known long before their discovery, since 1969. The toxic concentration of that substance (IC_50_) against the tumor cells L-1210 was very low (0.5 ng/ml), and in microgram doses (per one kg of test animal weight) it demonstrated a high antitumor activity against different types of mouse cancer.

Multi-stage total syntheses of that substance could not provide researchers with a sufficient amount of material for bioassay. For instance, the total yield in the first such synthesis was less than 1% [9]. As a result, methods of ascidian cultivation were developed, and submarine plantations were created near the coast of Spain. However, that method of production of the basic biological material was also rather inappropriate due to significant variations in the content of ecteinascidins, which were used in clinical testing, in the ascidians cultivated. Finally, after long research, scientists managed to obtain this alcaloid by chemical transformations from antibiotic cyanosafracin B, which is produced with a good yield by the terrestrial bacterium Pseudomonas fluorescens [10].

The mechanism of biological action of the trabectidin active substance, ecteinascidin-743, on tumor cells is related to its ability to penetrate the DNA's minor groove and to alkylate guanine residues [11]. Moreover, trabectidin causes programmed death (apoptosis) of tumor cells and intensifies the antitumor action of some well-know drugs (doxorubicin, paclitaxel (taxol), etc.). In spite of the fact that trabectidin was approved only for the treatment of soft tissue sarcoma, it showed good results in the course of clinical testing for the treatment of other types of malignant tumors. Not long ago, the Pharmamar Company (Spain) that has created trabectidin sold the license to Jonhson&Jonhson/Ortho Biotech to promote that drug on the American market.

A series of other marine natural products possessing extremely high cytotoxicities against tumor cells like spongistatin and ecteinascidin are being studied now as potential antitumor drugs and are subject to different stages of clinical and preclinical testing [12, 13] (Table 1).

**Table 1:** Certain marine natural compounds are potential anticancer drugs

Compound	Biological source	Chemical nature	Action mechanism	Company	Status
Bryostatin-1 (**7**)	Bryozoan	Poliketide	Inhibitor of protein kinase	GPC Biotech	Phase 2 clinical trials
Dolastatin-10 (**8**)	Mollusc	Peptide	Inhibitor of microtubeles formation	NCI-Knoll	Phase 2 clinical trials of its derivative TZT-1027
HTI286 (**9**)	Sponge	Tripeptide	Inhibitor of microtubeles formation	Novartis	Continuation of clinical trials
Discodermalide (**10**)	Sponge	Poliketide	Inhibitor of microtubeles formation	Novartis	Phase 2 clinical trials
Cryptophycin (**11**)	Cyanobacterium	Cyclic depsipeptide	Inhibitor of microtubeles formation	Eli Lilly	Taken out from phase 2 clinical trials
Aplidin (**12**)	Ascidian	Cyclic depsipeptide	Causes oxidative stress in cells	PharmaMar	Phase 2 clinical trials
Eribulin mesylate (**13**)	Sponge	Polyether derivative	Inhibitor of microtubeles formation	Esai Company	Phase 3 clinical trials
Squalamine (**14**)	Shark	Steroid	Inhibitor of angiogenesis	Genaera	Taken out from phase 2 clinical trials
Kohalalide F (**15**)	Mollusc	Cyclic depsipeptide	Active on lysosomal level	PharmaMar	Phase 2 clinical trials
Salinosporamide A (**16**)	Marine bacterium	Lactam-lactone derivative	Proteosome inhibitor	Nereus	Phase 2 clinical trials

For instance, bryostatin-1 (7) (Fig. 2), the 26-membered macrocyclic lactone from the bryozoan Bugula neritina, a fouling organism that grows in thick colonies on pier pilings and docks in the World Ocean, was detected by Pettit and co-workers from Arizona State University after several samplings of the biological material. Its structure was elucidated with the help of X-ray analysis. The compound (7) has 11 asymmetric centres, and it is hardly probable it will be obtained with a sufficient yield by organic synthesis in the years to come. Its content in bryozoan is insignificant (0.00001%), but scientists have managed to obtain 18 g of bryostatin for clinical and preclinical testing from 10,000 gallons of bryozoan collected. This substance has been determined to be a modulator of protein kinase C, a stimulator of the immune system, and an inductor of cell differentiation. It intensifies the antitumor action of some drugs but causes myalgias as a side effect. Currently, this drug is being tested in combination with paclitaxel, vincristine, ara-C, etc. (Table 1).

Dolastatin 10 (8) (Fig. 2) was discovered after an expedition by Professor Pettit to the Mauritius Island in 1972, when they collected the marine nudibranch Dolabella auricularia and discovered the high antitumor activity of its extracts. To obtain the first milligram of the extract's active component - compound (8) - the scientists had to collect again a giant amount of that rare mollusk (about 2 tones) due to a low content of the required substance. Dolastatin 10 appeared to be a linear pentapeptide with residues of four previously unknown amino acids: N,N-dimethylvaline, dolaisoleucine, dolaproine, and dolaphenine [14]. In 1989, researchers carried out a total synthesis of that peptide, which confirmed the structure suggested and established its absolute stereochemistry [15]. Dolastatin 10 is extremely toxic against tumor cells, and its semitoxic concentration (IC_50_) against cells of limphocytic leukemia P388 is 4.5x10^-5^ μg/ml. However, at the first and second stages of clinical testing its high antitumor activity was not confirmed. A while ago, the clinical testing of dolastatin was discontinued.

On the other hand, attempts to create a dolastatin-based antitumor drug were not abandoned. It has been discovered that the synthetic dolastatin derivative TZT-1027, in which the dolaphenine aminoacid is replaced with the phenylalanine group, just like dolastatin, is a strong inhibitor of tubulin polymerization, stops the division of cancer cells in very low concentrations, and reduces blood supply to tumors (inhibits angiogenesis). Currently, TZT-1027 (soblidotine) is undergoing clinical testing in Japan, Europe, and the USA for the treatment of solid tumors, including those resistant to other drugs [16].

Hemiasterlin (9) (Fig. 2) is a tripeptide that was extracted for the first time from the deep-water sponge Hemiastrella minor by Kashman and his co-workers in 1986 [17]. Its synthetic analogue HTI-286, with a phenyl substituent instead of N-methylindol, appeared to be more active and, in nanomolecular concentrations, inhibited cell division binding with the monomeric units of tubulin and complicating its polymerization [18]. In preclinical testing, it showed good activity against tumors resistant to paclitaxel, one of the best antitumor drugs used currently. However, the clinical testing did not confirm that it was active in the case of patients in terminal stages of cancer. Recently, scientists demonstrated the antitumor action of this drug on androgen-dependent tumors, which has inspired renewed interest in further clinical studies of HTI-286 [19].

**Fig. 3.**
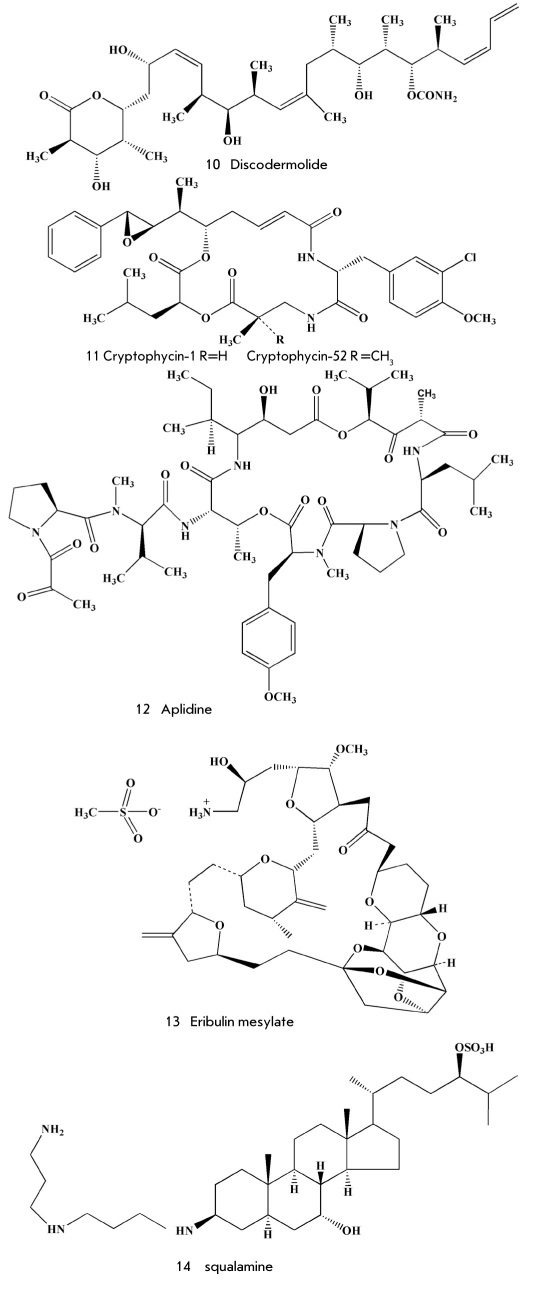
Some compounds tested as active substances of antitumor drugs

Discodermolide (10) (Fig. 3) was isolated by scientists of the Harbor Branch Oceanographic Institute (Florida, USA) from the rare deep-water sponge Discodermia disollata collected in the Bahamas at depths of up to 300 m using a submarine. The chemical structure of the compound (10) was elucidated with the help of a thorough analysis of NMR spectra and X-ray analysis, and it was confirmed by syntheses of discodermolide itself [22] and its antipode (-)-discodermolide [21], which in contrast to the natural (+)-isomer appeared to be much less active as a potential antitumor agent. Actually, natural discodermolide was able to stop the development of tumor cells at the G2/M phase of the cell cycle in concentrations of 3-80 nM, whereas the (-)-isomer was 2-20 times weaker. The drug appeared to be a much stronger inhibitor of tubulin polymerizations than paclitaxel; moreover, their combined action was stronger than the action of each one of those agents. After multiple improvements of different variants of the multistage discodermolide syntheses, researchers of the pharmaceutical company Novartis managed, finally, to obtain 20g of that substance, to complete its preclinical testing in 2004-2005, and to start clinical testing. To date, this testing has been discontinued. In spite of the fact that this drug is relatively low-toxic for patients, it remains insufficiently effective. Nevertheless, it may be used in combination with other antitumor drugs [13].

Tubulin-binding agents also include cryptophycins and related compounds. Cryptophycins are depsipeptides produced by cyanobacteria Nostoc spp. [23]. They got their name for strong inhibitory activity against the pathogenic bacteria Cryptococcus spp. However, their antitumor properties have attracted even more attention. For instance, cryptophycin-1 (11) (Fig. 3) is toxic against tumor cells in concentrations of 1-10 pg/ml. Complete synthesis of cryptophycin-1 [24] has made it possible to pinpoint its structure and allowed the Eli Lilly Company to start the creation of a cryptophycinbased antitumor drug. In particular, a synthetic analogue of cryptophycin-1, the so-called cryptophycin-52, has proved more active against tumor cells than vinca peptides and paclitaxel by 40 and 400 times, respectively. However, in clinical testing it has appeared to be highly toxic for patients. Testing had to be stopped in the end of the 1990s. Later, new derivatives - cryptophycin-309 and -249 - were obtained, which are now undergoing the final stage of preclinical testing [13].

Several highly active depsipeptides from ascidians, including didemnin B from Trididemnium solidum [25], were intensely studied for many years as potential antitumor drugs. However, in the mid 1990s, the clinical investigations of didemnin B were discontinued due to significant neuromuscular toxicity and insufficient effectiveness for patients in terminal stages of cancer. However, its analogue aplidin (12) (dehydrodidemnin B) (Fig. 3) from the Mediterranean ascidian Aplidium albicans initiates oxidative stress with the following apoptosis in tumor cells [26]. Aplidin is also an inhibitor of angiogenesis and disturbs blood supply to tumors. In spite of the fact that aplidin is at the second stage of clinical testing as a drug for myeloma treatment, a good method to produce it has not been developed yet, because neither the technology for the corresponding ascidian cultivation, nor an appropriate synthesis for the production of a sufficient amount of this substance have been elaborated [13].

In 1986, Uemura and Hirata isolated several minor metabolites called halichondrins from the sponge Halichondria okadai [27]. Those compounds were strong inhibitors of tumor cell development (IC_50_ 10^-9^ M), bound to tubulin on the same site as the vinca peptides applied in clinical treatment, and were selected for the further investigation of their antitumor properties. However, it was rather difficult to produce halichondrins in sufficient amount. Due to a complex structure, the total synthesis of halichondrin B developed in 1992 [28] consisted of 90 stages and could not solve that problem. Almost at the same time, New Zealand scientists discovered new source of halichondrins, the deepwater sponge Lissodendoryx n. sp.1. A ton of that sponge was obtained by dredging. Moreover, plantations of Lissodendoryx were created in shallow waters in New Zealand, though the content of the target agents was much lower in the cultivated tube than in the wild one [29]. Those efforts made it possible to obtain 310 mg of halichondrin B and to begin clinical testing in 2002. Then, Japanese scientists, in collaboration with the Esai Company, found out that a much simpler derivative of halichondrin - eribulin mesylate (13) (Fig. 3) - was characterized by the same biological activity. Currently, eribulin mesylate is in the third stage of clinical testing as a potential drug for the treatment of breast cancer [13]. Moreover, it is being tested for the treatment of prostate cancer and sarcoma.

Squalamine (14) (Fig. 3), a water-soluble aminosteroid, was extracted from the liver of the shark Squalus acanthis in 1993. This substance displays strong antimicrobial action [30, 31]. Later, using different types of mouse cancer, squalamine was found to inhibit angiogenesis and to stop the growth of tumors [32]. The Genaera Company organized pharmacological investigations of squalamine, but at the second stage of clinical testing (pulmonary and ovarian cancers), its antitumor properties were found to be insufficient. Nevertheless, squalamine was found to intensify the therapeutic effect of paclitaxel and carboplatin, inhibiting some growth factors, for instance VEGF, and causing a decrease in the amount of blood vessels around the tumor and apoptosis of tumor cells. Moreover, it was established that its physiological effects could be useful in the treatment of diseases characteristic of elderly people and related to vision disorders (macular degeneration) [33, 34].

**Fig. 4.**
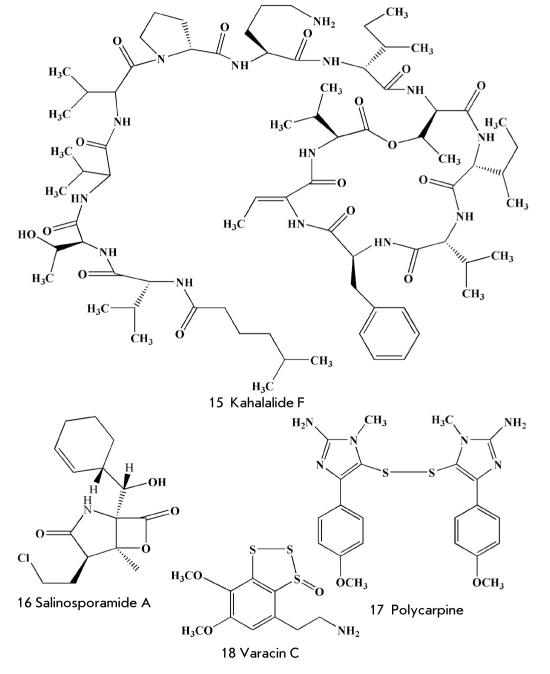
Some compounds investigated as active substances of antitumor drugs

The investigation of the mollusc Elysia rufescens under the guidance of Scheuer at the Hawaiian University in the USA led to the discovery of several new high-active depsipeptides, including kohalalide F (15) (Fig. 4) [35]. This mollusc feeds on the algae Bryopsis spp., the real producers of kohalalide. The mollusc accumulates this biologically active substance as a chemical protective means against predators. Moreover, the kohalalide content in the mollusc is 5,000 times higher than in the algae. After the solid-phase synthesis of that peptide, its structure and relative stereochemistry were corrected [36] and the PharmaMar company began the preclinical and then clinical investigation.

Kohalalide induces the formation of vacuoles in some tumor cells and stimulates lysosomes. It is several times more toxic against tumor cells than against healthy cells [37]. In spite of the fact that the mechanism of the kohalalide's antitumor action has yet to be pinpointed , currently, it is in the second stage of clinical testing for the treatment of solid tumors resistant to other substances [13].

Salinosporamide A (16) (Fig. 4) was isolated in 2003 by Fenical and co-workers (Scripps Institution of Oceanography, California, USA) from the salt-tolerant marine bacterium referred to as a new class of bacteria-actinomycetes called Salinispora [38]. It inhibits p26 proteosomes [36]. Not long ago, the pharmaceutical company Nereus Pharmaceuticals (USA) completed the first stage of clinical testing of that substance coded as NPI-0052 for the treatment of multiple myeloma [13].

In addition to the above-mentioned substances, several other marine natural products have been clinically studied as potential antitumor agents: peptide cematidine from moluscs, peptide ILX-65 similar to dolastatin, and tripeptide E-7974 from the sea sponge inhibiting polymerization of tubulin [5, 13, 40] (Knoll Company); derivative of aminoacid LAF389 being an inhibitor of methionine-aminopeptidase (Novartis); synthetic analog of sponge cerebroside KRN 7000 characterized by immunostimulatory and strong antitumor action on patients who retained a high level of NK cells. Prospects for the further clinical study of these substances are unclear.

In Russia, alkaloids - polycarpin (17) (Fig. 4) [41] and varacin C (18) [42], characterized by high toxicity against tumor cells, were isolated from ascidians. Cytotoxicity and activity in the acid environment of varacin C (18) are higher than those of well-known doxorubicin: that is why varacin is quite selective as regards tumors, in comparison to normal tissues [43]. In fact, some tumors are known to acidify themselves due to elevated glycolysis. Polycarpin and its numerous synthetic analogues cause apoptosis of tumor cells, intensifying the phosphorylation of protein p53 at the aminoacid residue Ser-15 [44]. However, they have appeared to be rather toxic to animals. Varacin C was synthesized shortly after isolation [43], and a while ago, in Russia, scientists began the synthesis of its analogues and obtained several high-active compounds promising for further investigation as pharmaceutical leads [45].

Hence, most of the substances selected for preclinical and clinical testing are strong inhibitors of tubulin polymerization. Moreover, they include inhibitors of protein kinase C (bryostatin), other enzymes (LAF-389), inhibitors of proteosomes (NPI-0052), agents interacting with DNA (Yondelis), inhibitors of angiogenesis (aplidin, kohalalide, and squalamine), and substances with an undetermined mechanism of action. Taking into consideration the wide diversity in the structures of highly active marine metabolites and the different mechanisms through which they exhibit antitumor action, there is confidence that further efforts aimed at creating antitumor drugs on the basis of marine natural products will be successful.

## Marine Natural Products with Analgesic Properties

The first analgesic drug based on marine natural products was called ziconotide (prialt). It was created after twenty years of investigating toxins from predatory moluscs-gastropods belonging to the Conus genus. In the end of 2004, this compound under the commercial name "prialt" was approved for production and clinical use in the USA, and a few months later, in Europe. The name "ziconotide" is more often used for its active substance, ω-conotoxin, obtained with the help of peptide synthesis.

Cone snails, more than 300 species are known, use small fish for food, which snails catch by a harpoon connected by a special channel to their poison gland. The snails' glands biosynthesize hundreds of different peptide toxins [46], which immobilize a victim by affecting the neuromuscular transmission in its organism.

**Fig. 5.**
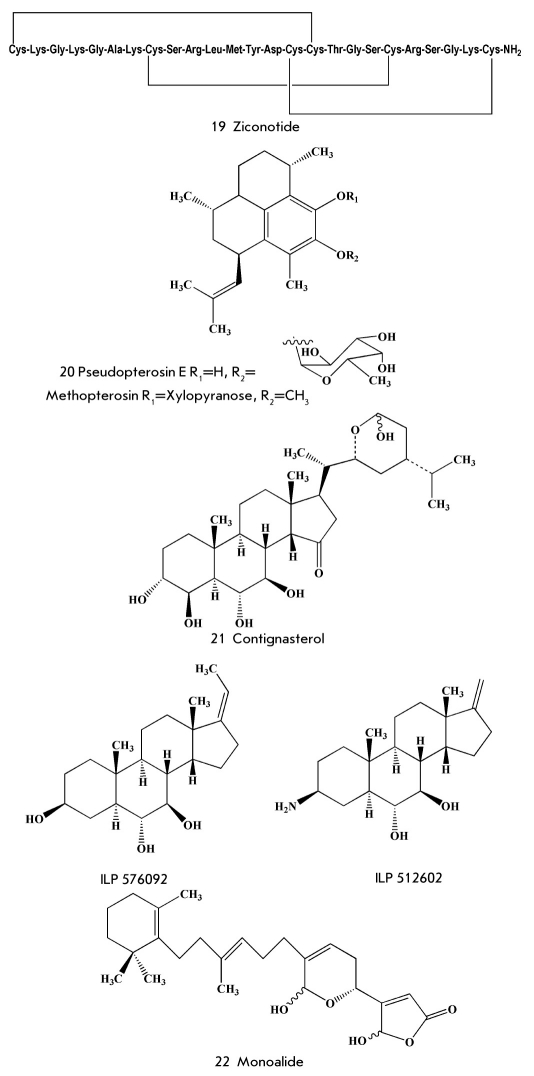
Some compounds investigated as analgesic and anti-inflammatory agents

Understanding of the structure of some toxins from different species of cone snails was followed by the synthesis of thousands of their analogues. However, pharmacological trials showed that one of the natural toxins, rather than their synthetic derivatives, was of top interest as a potential drug. That toxin was named ω-conotoxin MVIIA. ω-conotoxin is a linear peptide composed of 25 aminoacid residues, which was isolated for the first time from the Pacific mollusc Conus magnus. Six cysteine residues form three disulphide bridges in this compound [47, 48]. The disulphide bridges provide ω-conotoxin with a well-formed and unique space structure, as well as the ability to specifically block the work of N-type voltage-sensitive calcium channels. As a result, the toxin efficiently inhibits the transmission of the pain signal (Kd=9 pM). Clinical investigations of synthetic ω-conotoxin were carried out by the pharmaceutical company Neurex (branch Elan Pharmaceuticals). As an analgesic it appeared to be 1,000 times stronger than morphine [49]. Those investigations showed its high efficiency in the inhibition of pain, including phantom ones. In contrast to morphine, ziconotide (19) (Fig. 5) did not cause hallucinogenic effect and addicting property effect and does not cause addiction [50].

Several other conotoxins are now at different stages of investigation as potential drugs. Clinical trials of some compounds of that class were discontinued due to undesirable side effects: for instance, the AM-336 peptide-based drug, which was developed by the AMRAD Company for the treatment of chronic pains.

Recently discovered groups of conotoxins, which specifically block α1−adrenoreceptors, are good model compounds for the creation of new analgesic medical products on their basis [51].

## Anti-inflammatory and Wound-Healing Marine Natural Products

Pseudopterosins, for instance pseudopterosin E (20) (Fig. 5), are characterized by strong anti-inflammatory action. Pseudopterosins belong to the group of diterpene glycosides isolated by Fenical and co-workers from the Caribian gorgonian coral Pseudopterogorgia elisabethae in the end of the 1980s [52]. The anti-inflammatory action of pseudopterosins is stronger than that of the well-known anti-inflammatory drug indomethacin. They influence the biochemical transformation of arachidonic acid, decreasing the synthesis of eicosanoids [53]. Estee Lauder has created a cosmetic cream for facial skin for commercial realization on the basis of partially purified extracts of P. elisabethae, containing pseudopterosin E. To ensure production of the cream with the required amount of pseudopterosins, scientists collected a lot of gorgonian corals along the coast of the Bahamas Islands and, then, studied the regeneration of corals after the removal of some parts of their colonies. Two other ways to produce pseudopterosin were developed to decrease the damage to underwater biocenoses: their aglycons were synthesized by several methods [54-56] and, then, glycosylated: moreover, scientists have discovered new biotechnological ways to produce pseudopterosin from farnesol pyrophosphate under the action of enzymes extracted from P. elisabethae [57].

The synthetic derivative of pseudopterosins called methopterosin (OAS 1000) was subjected to clinical testing as an anti-inflammatory agent for the treatment of contact dermatitis. However, those trials were not completed due to the bankruptcy of the OsteoArthritis Sciences Inc. Company [34]. Later, that substance was subjected to the second stage of clinical testing as a wound-healing agent by Tyrosin Group Inc. Company (USA).

Contignasterol (21) (Fig. 5) [58], an unusual steroid from the sponges Petrosia contignata, was studied under the code IZP 94005 as an anti-inflammatory agent. The structure of contignasterol is characterized by a cis-junction of the C and D cycles. In contrast to drugs with analogous action, this compound (21) is not an inhibitor of A2 phospholipase, but it inhibits the excretion of histamine by leukocytes and is referred to as a leukocyte-selective anti-inflammatory agent [59]. Inflazyme Pharmaceutical Ltd. and Aventis Pharma (USA) were jointly developing a new drug on the basis of contignasterol. However, because of its extremely complicated structure, contignasterol was modified and replaced with the simpler, but highly active, synthetic analogues IPL 576092 and IPL 512602 [60, 61]. This latter compound underwent two stages of clinical testing for the treatment of asthma. In 2004, cooperation between the two companies ended and Inflazyme chose to work independently: several more promising derivatives were obtained, while the testing included not only asthma, but also skin and eye disorders [34]. However, after the sale of this project in 2008 to Orexo Pharmaceuticals Company, information about further investigation of the substance has not appeared.

**Fig. 6.**
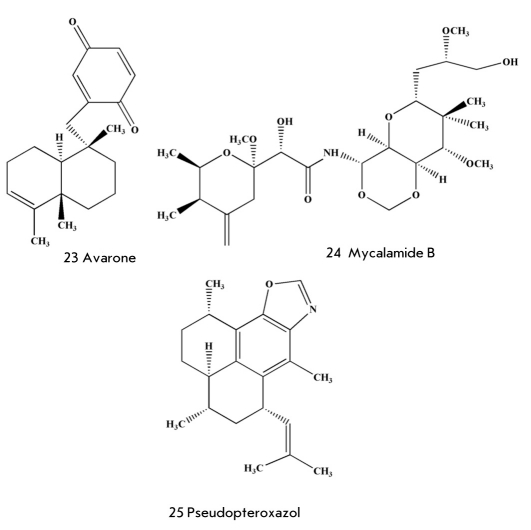
Marine natural antiviral compounds

One more terpenoid - monoalide (22) (Fig. 6) - was extracted from sponges Luffariella variabilis [62] by Scheuer and co-workers in 1980. Monoalide has two hidden aldehydic groups (hemiacetal and as a γ-lactone derivative) which react with the amino groups of lysine residues on the surface of the substratum binding site in A2 phospholipase. As a result, monoalide (22) inhibits this enzyme and the hydrolytic elimination of arachidonic acid from prospholipide, demonstrating anti-inflammatory properties. The strong anti-inflammatory action of monoalide [63, 64] has attracted the attention of Allergen Pharmaceutical Company (USA). The company secured a license for the drug and carried out two stages of clinical testing of monoalide as a drug for the treatment of psoriasis. However, problems with the low transport of the active substance through a patient's skin led scientists to stop further clinical investigations. At the same time, monoalide is released as a biochemical reagent, a specific inhibitor of A2 phospholipase. Moreover, scientists continue to try to obtain such a derivative of monoalide that will be devoid of its disadvantages, with the help of organic synthesis.

The Pacific Institute of Bioorganic Chemistry, Far East Division of the Russian Academy of Scientists, created a new drug called Collagenase KK based on a complex of collagenolytic proteases from the Kamchatka King crab Paralithodes chamtschaticus. After preclinical and clinical testing, Collagenase KK was approved for production and use in Russia. The drug was recommended for fermentative wound cleansing in case of pitting, necrosis, chilblains, gangrene, chronic osteomyelitis, and varicose ulcer [65-67]. Experience in the clinical application of Collagenase KK after the release of the first batches shows that this drug, in addition to the abovementioned areas of medical application, may be useful for the treatment of some other diseases and post-surgery complications. For instance, Collagenase KK was successfully used in endoscopic and plastic surgery and for the treatment of foul peritonitis in children. Collagenase KK may also be used for the destruction of collagen in commissures.

## Marine Natural Compounds with Antiviral and Antimicrobial Properties.

After the discovery of arabinonucleosides characterized by antiviral and antitumor properties in the beginning of the 1950s and after the following synthesis of some nucleoside derivatives, which became the biologically active substances of antiviral drugs, the search for new marine antiviral drugs has been in progress. Compounds with such activity were found among terpenoids, steroids, alkaloids, aliphatic and aromatic derivatives, peptides, polysaccharides, and other secondary metabolites extracted from different marine organisms [68-71].

AIDS remains one of the most dangerous viral diseases affecting a great number of people. The number of people suffering from AIDS approximates 50 million and increases every day by 16 thousand people. By the early 2003, more than 150 highly active marine metabolites were found in the course of testing against HIV [69, 70]. Edible algal polysaccharides, in particular fucoidans, carrageenans, and others, inhibit the penetration of HIV into human mononuclear cells. Some of them inhibit virus replication in very low concentrations (0.1-0.01 μg/ml) and intensify the antiviral action of azidothymidine. However, some specialists believe that their antiviral effects are the result of nonspecific interaction either with viruses, or with cells, while these substances themselves poorly penetrate biological fluids [70]. Hence, the question as to whether these substances can be considered as additional agents that in future may be used for the treatment of AIDS patients remains a matter of debate.

Peptides from some marine invertebrates are one more perspective group of antiviral substances. For instance, peptides composed of 17-18 amino-acid residues from horseshoe crabs Tachypleus tridentatus and Limulus polyphemus are characterized by strong antiviral effect against HIV-1. Analogous, but simpler in structure compounds were synthesized in the course of a project aimed at creating a new drug on the basis of those peptides. One of the peptides (T144) had IC_50_=2.6 nM against HIV-1 at low cytotoxicity (IC_50_=44.6 μM) [72, 73, 69]. The project aimed at creating a T144-based antiviral drug has a fair chance of success.

Among the highly active low-molecular antiviral marine substances it is important to note the above-mentioned didemnin B from ascidians, which when injected every day in a dose of 0.25 μg/kg to mice contaminated with a lethal dose of Rift Valley fever helped save 90% of them. Some hope is related to a relatively simple terpenoquinone avarone (23) (Fig. 6) and similar compounds from the sponges Dysidea, which inhibit reverse transcriptase from HIV-1 and the virus itself in a concentration of 0.1 μg/ml [70]. Mycalamide B (24) (Fig. 6) from the New Zealand Mycale sp. may be cited as another example of antiviral metabolite from sponges. Mycalamide B is a strong inhibitor of protein synthesis, which shows inhibiting activity in a dose of 2 ng/band when tested in vitro for action on different viruses [70]. The creation of drugs on the basis of these and other highly active compounds is slowed down by the high toxicity of some of them and low accessibility. However, these and other highly active marine substances are good models for syntheses of new less toxic, but highly active, antiviral agents.

**Fig. 7.**
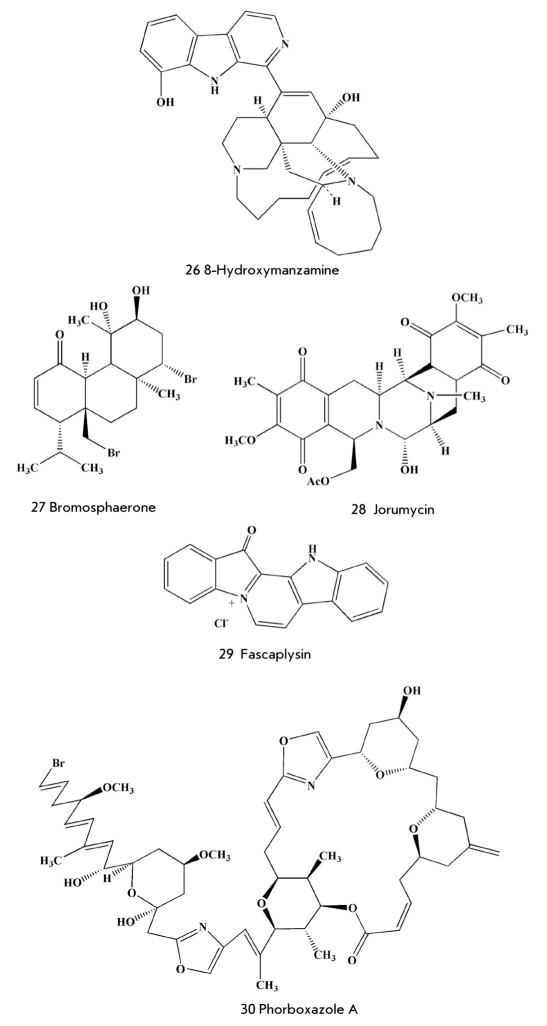
Marine natural anti-infectious agents

Some of the numerous marine antimicrobial compounds displayed high activity against the tuberculosis bacterium Mycobacterium tuberculosis. For instance, pseudopteroxazol (25) (Fig. 6), benzoxazole diterpene alkaloid from the gorgonian coral Pseudopterogorgia elisabethae, inhibits the growth of this mycobacterium by 97% in a concentration of 12.5 μg/ml in the absence of toxic effects [71], while (+)-8-hydroxymanzamine (26) (Fig. 7) from the sponge Pachypellina sp [71] has a minimum inhibiting concentration of 0.91 μg/ml. Manzamine is even more active against the protozoa Toxoplasma gondii (IC_50_=0.054 μg/ml), an infectious agent which causes such extremely dangerous (especially for pregnant women and children) diseases as toxoplasmosis. Manzamine is not used in medicine due to its low accessibility.

Diterpenoid bromosphaerone (27) (Fig. 7) from the red alga Sphaerococcus coronopifolius (IC_50_=0.078 μg/ml), dimeric isoquinoline alkaloid jorumicin (28) (Fig. 7) from the Pacific sea hare Jorunna funebris (IC_50_=0.050 μg/ml), and some other marine metabolites [71] are highly active against Staphylococcus aureus.

However, most of these substances, as well as fascalkaloid fascaplysin (29) (Fig. 7), macrolide forboxazole A (30) (Fig.7) and others characterized by strong antifungal action, have appeared too toxic for use in clinical testing.

## Marine Natural Products with Other Biological Effects

**Fig. 8.**
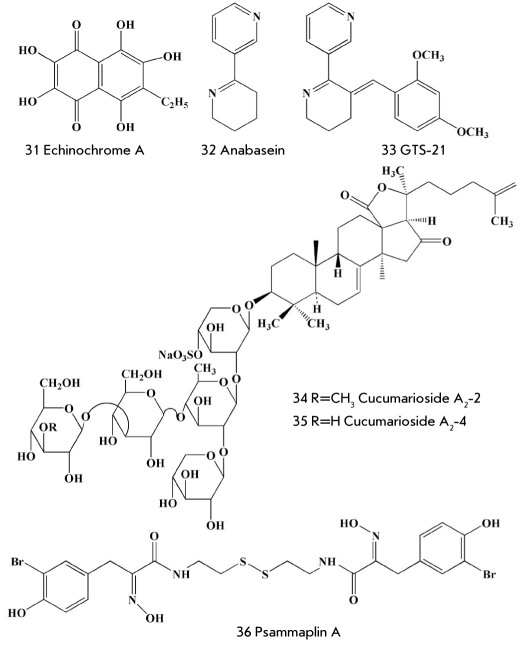
Other physiologically active marine natural compounds

Two new drugs - Histochrome for ophthalmology and Histochrome for cardiology - have been created in the Pacific Institute of Bioorganic Chemistry, Russian Academy of Sciences, on the basis of sea urchin pigments characterized by antioxidant, antimicrobial and anti-inflammatory properties and then approved for production and use in Russia [74]. Histochrome for ophthalmology has proved highly effective against traumas and blood strokes of different origins and against some other eye diseases. Histochrome for cardiology is a cardioprotector reducing by half the necrosis zone formed due to acute myocardial infarction. Histochrome for cardiology commonly assigned as drop infusion 10 minutes before the thrombolytic therapy decreases the frequency of extrasystoles. Moreover, this drug decreases the number of complications after open-heart surgery. The major constituent active substance of both drugs is the well-known pigment echinochrome (31) (Fig. 8). Scientists have found an easily accessible natural source of echinochrome (the sand dollar Scaphechinus mirabilis) and have developed new methods of isolation and synthesis of this pigment with a high yield [47].

The clinical application of Histochrome drugs has demonstrated their advantages relative to the analogous drugs used previously. The data obtained on the Histochrome for ophthalmology are of special interest. Histochrome for ophthalmology has been established to be successful in the treatment of hemophthalmos, different child eye pathologies, cataract, as well as in eye surgery.

In 1971, a toxin called anabasein (32) (Fig. 8) was extracted from the sea worm hoplonemertea [75]. The synthesis of numerous anabasein analogues has led to the creation of several highly active compounds, including the pharmaceutical lead GTS-21 (33) (Fig. 8), which has displayed cytoprotector properties and improved memory in test animals. Competing with the natural ligands, GTS-21 binds to α4β and the α7-subtypes of nicotinic receptors; the latter is considered to be important in control of β-amyloid neurotoxicity. Florida State University sold to the Japanese company Taicho the license for those substances, and they have organized clinical testing in Europe and the U.S. The first stages of testing on patients-volunteers showed the significant cognitive effects of GTS-21 [76]. Currently, substances of this series are being studied as potential drugs for the treatment of Alzheimer's disease.

Holothurians (sea cucumbers), invertebrates belonging to the Holothurioidea class of the phylum Echinodermata, have always attracted the attention of people in Eastern Asian countries due to their biologically active substances. Edible animals of that class (trepangs) are thought to have healing properties, including stimulation, wound-healing, and other useful effects. However, the substances responsible for the biological activity of trepangs have yet to be studied.

In the course of a multi-year investigation, scientists from the Pacific Institute of Bioorganic Chemistry, Russian Academy of Sciences, have collected about 50 species of holothurians in different parts of the World Ocean and obtained more than 100 new physiologically active triterpene glycosides from their extracts [76]. Some of them have turned out to be strong modulators of cellular immunity. These substances serve as a basis for the development of a potential drug called cumaside, which contains the agents (34, 35) (Fig. 8) as an active substance [77]. This drug, in very low concentrations, increases the resistance of test animals to bacterial infections and radiation, stimulates the phagocytic and bactericide activities of macrophages, inhibits tumor growth and intensifies the action of antitumor drugs, without displaying any toxic properties [78, 79]. Cumaside will be subjected to clinical testing in the nearest future.

## Conclusion

Marine natural products are quite diverse. Every year, the number of known marine natural products increases (between 2007 and 2008, almost by 1,000 compounds every year) [80]. Their biochemical diversity is a result of the high biological diversity in the seas and oceans. According to different estimates, our planet is inhabited by several million species of marine microorganisms, as well as about a million of yet undescribed species of marine invertebrates and other marine organisms.

The investigation of marine natural products has led to the creation of a series of highly efficiency medical products, including antitumor and antiviral drugs (Arabinocytosine, Arbinoadenosine, Trabectidin), the analgesic drug Prialt, two Russian drugs of the Histochrome series, which are able to decrease the necrosis zone after myocardial infarction and to treat the consequences of eye blood stroke of a different ethiology, as well as the Russian burn-treating drug Collagenase KK. More than 40 antitumor, anti-inflammatory, immunostimulatory, and other pharmaceutical leads are at different stages of preclinical and clinical testing. This all became possible thanks to the physiological activity of some marine natural products. Among them are the most potent non-protein toxins (palytoxin, maitotoxin), the most effective inhibitors of tumor cells development (spongistatin, etc.), the strongest analgesic compounds (the toxins of cone snails), and other extremely active substances.

It appears that many marine secondary metabolites extracted from sponges, ascidians, and other marine invertebrates are biosynthesized by symbiont microorganisms [80]. Only 1% of such microorganisms can grow in an artificial environment and may be cultivated. An example is the extraction of the bacterium Micromonospora sp from the deepwater sponge by American scientists. It produces manzamine and 8-hydroxymanzamine. As was mentioned before, these substances show very promising anti-parasitogenic and antituberculous activity, but they cannot be obtained in a large amount from the sponges. It is of interest that the biosynthesis of physiologically active alkaloids in this bacterium is possible only in a special environment, while the standard cultivation is not appropriate for their formation [5]. Recently, Fenical and his co-workers selected a medium for the cultivation of marine microorganisms with regard to conditions characteristic of their habitats. As a result, they have managed to cultivate a range of marine actinomycetes and to obtain a series of new secondary metabolites from them [81]. As follows from their investigation of the related species belonging to the new class Salinispora, the biosynthesis of secondary metabolites in marine microorganisms is often not strain-specific, but species-specific, which makes these microorganisms an appropriate and reproducible source of highly active compounds. In the opinion of Newman and other scientists from the National Institute of Cancer (USA), the study of bioactive substances from marine microorganisms has just begun [81].

In recent years, a new direction in the search for and study of marine natural products - marine metagenomics - has appeared. Within the framework of this direction, not individual genomes, but a group of genomes from any habitat, for instance from sponges, are analyzed and subjected to manipulations. In addition to the sponge genome, the genomes of numerous microorganisms, largely those hard to cultivate, are investigated. The transfer of big gene clusters to bacteria which are easy to cultivate, followed by the analysis of the metabolite products in some clones, may lead to the creation of producers of useful compounds [82, 83]. However, it is essential to have knowledge about the biosynthesis of these substances to solve the problem of the creation of useful natural products by this method. A while ago, scientists reported on the transcription of the genes responsible for the biosynthesis of such highly active marine metabolites as bryostatins and some metabolites extracted from sponges [83, 84]. The metagenome projects are being realized now on vast water areas (Monterey Bay, the Sargasso Sea) [85].

Some marine organisms are characterized by high productivity. For instance, the productivity of microalgae is higher than that of agricultural plants. They may be the sources not only of such useful for medicine substances as ω-3 fatty acids and carotenoids, but, likely, of other highly active compounds, as well as biofuel. It is necessary to select the most promising strains of these plants and to develop new-generation phytobioreactors to obtain biomass in an amount sufficient for the solution of these problems [86].

Finally, the study of biologically active marine natural compounds stimulates work aimed at the organic synthesis of these compounds, as well as that of their derivatives and analogues. As a result, extensive libraries of compounds, including those characterized by a higher activity than a substance's prototype, are created. For instance, in the course of the investigation of histone deacetylase inhibitors, the Novartis Company, in cooperation with the group of well-known chemist-synthesist Nikolaou, using the marine natural compound psammaplin A (36) (Fig. 8) as a prototype, obtained a library of 3,828 substances through organic synthesis; 6 of those substances were highly active against the methicillin- and vincamycin-resistant strains of Staphylococcus aureus [5].
